# The development and validation of the clinicians’ awareness towards cognitive errors (CATChES) in clinical decision making questionnaire tool

**DOI:** 10.1186/s12909-017-0897-0

**Published:** 2017-03-21

**Authors:** Keng Sheng Chew, Yee Cheng Kueh, Adlihafizi Abdul Aziz

**Affiliations:** 1 Department of Surgery, Faculty of Medicine and Health Sciences, Universiti Malaysia Sarawak, 94300 Kota Samarahan, Sarawak Malaysia; 2 Unit of Biostatistics and Research Methodology, School of Medical Sciences, Universiti Sains Malaysia, 16150 Kubang Kerian, Kelantan Malaysia; 3 Emergency Medicine Department, School of Medical Sciences, Universiti Sains Malaysia, 16150 Kubang Kerian, Kelantan Malaysia

**Keywords:** Validity, Reliability, Cognitive Errors, Clinical decision making, Exploratory factor analysis

## Abstract

**Background:**

Despite their importance on diagnostic accuracy, there is a paucity of literature on questionnaire tools to assess clinicians’ awareness toward cognitive errors. A validation study was conducted to develop a questionnaire tool to evaluate the *C*linician’s *A*wareness *T*owards *C*ognitive *E*rror*s* (CATChES) in clinical decision making.

**Methods:**

This questionnaire is divided into two parts. Part A is to evaluate the clinicians’ awareness towards cognitive errors in clinical decision making while Part B is to evaluate their perception towards specific cognitive errors. Content validation for both parts was first determined followed by construct validation for Part A. Construct validation for Part B was not determined as the responses were set in a dichotomous format.

**Results:**

For content validation, all items in both Part A and Part B were rated as “excellent” in terms of their relevance in clinical settings. For construct validation using exploratory factor analysis (EFA) for Part A, a two-factor model with total variance extraction of 60% was determined. Two items were deleted. Then, the EFA was repeated showing that all factor loadings are above the cut-off value of >0.5. The Cronbach’s alpha for both factors are above 0.6.

**Conclusion:**

The CATChES questionnaire tool is a valid questionnaire tool aimed to evaluate the awareness among clinicians toward cognitive errors in clinical decision making.

## Background

According to the Institute of Medicine’s report titled “Improving Diagnosis in Health Care (2015)”, diagnostic error is defined as “the failure to (a) establish an accurate and timely explanation of the patient’s health problem(s) or (b) communicate that explanation to the patient” [1]. Three broad categories of diagnostic errors have been identified by Graber et al. [2], viz., the no fault errors, system-related errors and cognitive errors [1, 2]. The category of no-fault errors is defined as errors caused by external factors outside the control of the clinician or the health care system. These include atypical disease presentation or the misleading information provided by the patients. The second category, i.e., system-related errors, are errors due to technical or organizational barriers such as weaknesses in communication and care coordination, inefficient processes and faulty equipment. The third category, i.e., cognitive errors (also known as cognitive biases), are errors due to poor critical thinking skills of the clinicians [3, 4]. Cognitive errors are deviations from rationality and may derail the clinicians into making diagnostic errors if left unchecked [5]. Although they may believe otherwise, studies have shown that clinicians are in fact, just as prone to commit cognitive errors as anyone else [6, 7].

Campbell et al. [8] have classified the common clinically important cognitive errors into six categories [8]. These categories are (1) “errors due to over-attachment to a particular diagnosis (examples of cognitive biases in this class include anchoring and confirmation bias)”, (2) “errors due to failure to consider alternative diagnoses (for example, search satisficing)”, (3) “errors due to inheriting someone else’s thinking (for example, diagnostic momentum and framing effect)”, (4) “errors in prevalence perception or estimation (for example, availability bias, gambler’s fallacy and posterior probability error)”, (5) “errors involving patient characteristics or presentation context (for example, cognitive biases: fundamental attribution error, gender bias)”, and (6) “errors that are associated with the doctor’s affect or personality (for example, visceral bias and sunk cost fallacy)” [8].

In a survey by MacDonald et al. [9] involving 6400 clinicians on diagnostic errors, the top three reasons for diagnostic errors cited have to do with cognitive errors [9]. A total of 75% of these clinicians cited atypical patient presentation (resulting in the doctors being misled to consider other diagnoses), 50% cited failure to consider other diagnoses while 40% cited failure to gather adequate history from patients [9].

Nonetheless, as important as these cognitive errors are, it is not known how many clinicians are aware of this. As pointed out by Prochaska et al. [10] in their Transtheoretical Model of Change, the first step towards behavioral change is known as contemplation. In the context of cognitive errors in clinical decision making, this is the stage where a clinician becomes acutely aware of the negative impact of cognitive errors on diagnostic accuracy as well as factors that increase the vulnerability of a clinician in committing such biases in clinical decision making. Once a clinician is in the contemplation stage, he or she would likely see the necessity to initiate steps towards the intended behavioral change (in this case, minimizing the risk of committing cognitive errors when making clinical decisions). This step is known as the preparation stage [10].

On the other hand, a person who is unaware of the problem sees no reason to take any action to change. This prior stage is known as the pre-contemplation stage [10]. A tool is therefore necessary to facilitate the transition from the stage of pre-contemplation to the stage of contemplation.

Despite the impact of cognitive errors on diagnostic accuracy, there is a paucity of literature on questionnaire tools aimed to assess the clinicians’ awareness toward cognitive errors. This paper describes the development and validation of a questionnaire purported to evaluate the *C*linician’s *A*wareness *T*owards *C*ognitive *E*rror*s* (CATChES) in clinical decision making. The purpose of this tool is to help create the awareness among clinicians who are in the pre-contemplation stage with the hope of moving them from this stage to the stage of contemplation in the Transtheoretical Model of Change. This tool can also be used as a pre-intervention material to supplement educational resources in teaching cognitive errors in clinical medicine (such as this resource in MedEdPORTAL [11]).

## Methods

### Participants

For content validation, based on the recommendation by Lynn [12], ten experts consisting of emergency physicians from Universiti Sains Malaysia were invited to determine the content validation and out of these ten, nine of them consented.

For construct validation, emergency physicians and emergency residents with a minimum of four years’ working experience in Hospital Universiti Sains Malaysia were identified as the participants. Using the rule of thumb of a minimum of five participants per item, a minimum of 30 participants were needed. Clinicians who were not residents pursuing a postgraduate degree in emergency medicine or clinicians with less than four years of working experience in the emergency department were excluded. The authors invited 35 of these emergency residents to participate in the construct validation and 31 of them responded. All nine emergency physicians who participated in the content validation also participated in this construct validation process. Hence, a total of 40 participants were recruited in this construct validation process.

### Materials

The questionnaire tool in this study is divided into two parts. The first part of the questionnaire (Part A) aimed to evaluate the awareness of clinicians towards cognitive errors in clinical decision making while the second part (Part B) aimed to evaluate the clinician’s perception towards specific categories of cognitive errors in clinical setting (Part B).

A preliminary version of the questionnaire was first developed by two authors (KS and AH) and checked by the third author (YC). For the development of Part A of this questionnaire, the Transtheoretical Model of Change [9] was used as the theoretical framework. Six items were generated in this preliminary version. The theoretical basis for each of the items is given in Table 1. Whereas, for Part B, the classification of cognitive errors used by Campbell et al. [10] was used to generate the preliminary list of categories of cognitive errors. Each category of the cognitive errors is defined as an item. A total of six items were generated.

**Table 1:** Preliminary list of items to evaluate the attitude of clinicians toward cognitive errors in clinical decision making

Item	Rationale of this item
Item no. 1 *“Cognitive errors in general have important impact towards clinical decision making in emergency medicine”*	This item is aimed to evaluate whether the clinician has any awareness towards the impact of cognitive errors in clinical decision making. Is the clinician in precontemplation stage or contemplation stage?
Item no. 2 *“Being aware of cognitive errors help me to be more careful in my clinical decisions”*	This item is aimed to evaluate whether the clinician believe that realize that by just being aware of these cognitive errors would improve the quality of his clinical decisions.
Item no. 3 *“Authority gradient discourage critical thinking and thus increase the vulnerability to commit cognitive errors”*	Authority gradient is defined as the gradient that exists between two individuals of different professional status, experience, or expertise that contributes to difficulty in exchanging information (Cosby and Croskerry, 2004). This item is aimed to assess the clinician’s perception on whether he or she believes that authority gradient discourages critical thinking on cognitive errors toward clinical decision
Item no. 4 *“Something, rather than nothing, can be done to minimize the risk of falling into these errors”*	To assess the motivation of the clinician towards change by minimizing the impact of cognitive errors in clinical decision making
Item no. 5 *“The understanding of cognitive errors and its impact on clinical decision making and patient safety should be made a component in emergency medicine curriculum in postgraduate training”*	To assess the motivation of the clinician towards change by minimizing the impact of cognitive errors in clinical decision making
Item no. 6 “*The understanding of cognitive errors and its impact on clinical decision making and patient safety should be taught at undergraduate level”*	To assess the motivation of the clinician towards change by minimizing the impact of cognitive errors in clinical decision making

### Procedure

This was a cross-sectional study conducted among clinicians (emergency physicians for content validation; emergency physicians and emergency residents for construct validation) from Hospital Universiti Sains Malaysia (HUSM). Convenience sampling was applied in recruiting the participants. Human Research Ethics approval was obtained from The Human Research Ethics Committee of Universiti Sains Malaysia before the study was commenced.

The content validity of the questionnaire (both Part A and Part B) was first determined by a panel of experts consisting of the emergency physicians in HUSM. These experts were briefed by one of the authors (AH) on how to respond to the relevance of the items, ranked in a Likert scale of four, ranging from “1 = not relevant at all” to “4 = highly relevant”. The experts were told to respond anonymously and that they were free to withdraw from the study at any time. The response sheets were passed to the experts to respond on their own and were collected back by author (AH) the following day. The document on the glossaries of terms were handed out and read out to the participants prior to starting the questionnaire.

After the content validation process, the construct validation of the questionnaire was determined. For the construct validation of Part A, participants were first briefed on how to respond to the items ranked in a Likert scale of five, ranging from “1 = strongly disagree” to “5 = strongly agree”. Participants were told to respond anonymously and that they were free to opt out at any time. A separate document on the glossaries of terms were handed out and read to the participants prior to starting the questionnaire. All participants responded individually in one sitting.

**Fig. 1:**
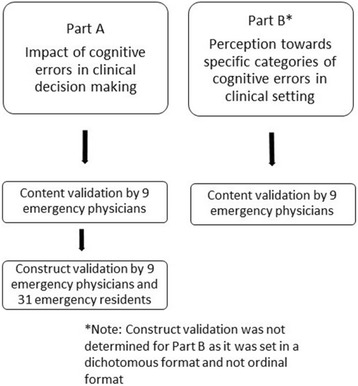
The sequence of content validation followed by construct validation

Since the purpose of this Part B is to identify the clinician’s perception towards the specific categories of cognitive errors in clinical setting, it was set in a dichotomous format (i.e., whether they are relevant or not relevant) and not in an ordinal format. As such, construct validation for this part was not determined. The sequence of content and construct validation is illustrated in Fig. 1.

### Statistical analyses

Exploratory factor analysis (EFA) was used to determine the construct validity of Part A of the questionnaire. Principal axis factoring was chosen as the extraction method. The initial run of the factor analysis was performed to determine the number of items to be extracted. An eigenvalue of more than 1 was chosen as the cut-off value to determine whether the numbers of factors to be fixed. Scree plotting was also performed to further verify the number of factors for extraction. Repeated runs of the factor analysis were then performed to determine the factor loadings of the items as well as to identify problematic items that may need to be removed. A cut-off point of 0.5 was used as the criteria in factor loading to determine whether an item is to be removed or not [13]. Whereas for communality (extraction), a value of >0.25 was set as the cut-off value to determine the need for item removal [14]. Promax oblique rotation was used. The internal consistency reliability of the item was determined by analyzing the Cronbach’s alpha coefficients. Cronbach’s alpha refers to the degree to which participants’ responses are consistent across the items within this questionnaire construct [15]. A cut-off point of Cronbach’s alpha >0.6 was set for this study for the criteria of a good degree of internal consistency [15]. The software SPSS version 22.0 for Mac was used for data analysis.

To evaluate the content validity of item relevance, the content validity index (CVI) and the modified kappa (κ) were used. The item relevance CVI (I-CVI) for relevance is defined as the proportion of the judges who rate the item with scores of 3 or 4 on a four-point Likert scale (with 1 = not relevant at all, 2 = somewhat relevant, 3 = quite relevant, and 4 = highly relevant) [12]. CVI value of 0.85 and above is considered as valid [12]. The modified kappa (κ) was computed in order to account for the possibility of chance agreement in CVI [16].

## Results

With regards to the content validity, the CVI values for all items were rated highly as valid in terms of their relevance in clinical settings. In terms of the values of their modified kappa (κ), all items were rated as “excellent” in terms of the validity of their relevance in clinical settings. The results of CVI for Part A and B are given in Tables 2 and 3 respectively.

**Table 2:** Content Validity of Item Relevance for Part A of Questionnaire

Item	N	A	P_C_	I-CVI		Evaluation of
Item no. 1 *“Cognitive errors in general have important impact towards clinical decision making in emergency medicine”*	9	9	0.00195	1	1	Excellent
Item no. 2 *“Being aware of cognitive errors help me to be more careful in my clinical decisions”*	9	9	0.00195	1	1	Excellent
Item no. 3 *“Authority gradient discourage critical thinking and thus increase the vulnerability to commit cognitive errors”*	9	8	0.01757	0.89	0.89	Excellent
Item no. 4 *“Something, rather than nothing, can be done to minimize the risk of falling into these errors”*	9	9	0.01757	1	1	Excellent
Item no. 5 *“The understanding of cognitive errors and its impact on clinical decision making and patient safety should be made a component in emergency medicine curriculum in postgraduate training”*	9	9	0.00195	1	1	Excellent
Item no. 6 *“The understanding of cognitive errors and its impact on clinical decision making and patient safety should be taught at undergraduate level”*	9	9	0.00195	1	1	Excellent

P_c_ is the probability of chance of occurrence. The formula for P_c_ is: N!/[A!*(N-A)!]*0.5^N^ where *N* the number of judges, *A* the number agreeing on good relevance. I-CVI is A/N

The formula for modified kappa statistic () = (I-CVI – P_c_)/(1 – P_c_), where P_c_ represents probability of a chance occurrence (Polit et al. [16])

Evaluation criteria for modified kappa (): = fair (0.40–0.59), = good (0.60–0.74) and = excellent (>0.74)

I-CVI should be 0.85 and above (Lynn 1986) to establish validity with a p < 0.05

**Table 3:** Content Validity of Item Relevance for Part B of Questionnaire

Item	N	A	P_C_	I-CVI		Evaluation of
Item no. 1 *“Cognitive errors due to over-attachment to a particular diagnosis”*	9	9	0.00195	1	1	Excellent
Item no. 2 *“Cognitive errors due to failure to consider alternative diagnoses”*	9	9	0.00195	1	1	Excellent
Item no. 3 *“Cognitive errors due to inheriting someone else’s thinking”*	9	9	0.00195	1	1	Excellent
Item no. 4 *“Cognitive errors in prevalence perception or estimation”*	9	8	0.01757	0.89	0.89	Excellent
Item no. 5 *“Cognitive errors involving patient characteristics or presentation context”*	9	9	0.00195	1	1	Excellent
Item no. 6 *“Cognitive errors that are associated with the doctor’s affect or personality”*	9	9	0.00195	1	1	Excellent

P_c_ is the probability of chance of occurrence. The formula for P_c_ is: N!/[A!*(N-A)!]*0.5^N^ where *N* the number of judges, *A* the number agreeing on good relevance. I-CVI is A/N

The formula for modified kappa statistic () = (I-CVI – pc)/(1 – pc), where P_c_ represents probability of a chance occurrence (Polit et al. [16])

Evaluation criteria for modified kappa (): = fair (0.40–0.59), = good (0.60–0.74) and = excellent (>0.74)

CVI should be 0.85 and above (Lynn 1986) to establish validity with a p < 0.05

**Fig. 2:**
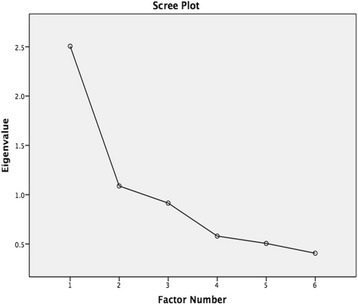
Scree Plot Showing The Two Factors Above The Point Of Inflexion

With regards to the construct validation using EFA on Part A, generally the Kaiser-Mayer-Olkin measure of sampling adequacy was found to be 0.74 which demonstrates a moderate degree of common variance shared among the items. The Bartlett’s test of sphericity was statistically significant (with chi-square statistics = 43.93, *p* < 0.05). This shows that there are correlations among the items based on the correlation matrix. Initial eigenvalue indicates that the first two factors (which has the eigenvalue >1) explain 60% of the total variance (42 and 18% respectively). Furthermore, 2 factors were shown to be above the point of inflexion of eigenvalue on the scree plot (Fig. 2). The number of factors was therefore, fixed at 2 for re-run of the analysis.

After 2 rounds of test re-run, two items out of the six were removed as they did not meet the minimum cut-off points of factor loading >0.5, and communality values of >0.25. In particular, item no. 3 *(“Authority gradient discourage critical thinking and thus increase the vulnerability to commit cognitive errors”)* was recognized as problematic with factor loadings of only 0.14 and 0.20 in both factors and its communality value (extraction) of 0.084 only. Item no. 4 *(“Something, rather than nothing, can be done to minimize the risk of falling into these errors”)* was also identified as problematic with factor loading of 0.481 in Factor 2 and a communality (extraction) value of 0.145.

After removal of the two items, the re-run of the principal axis analysis of the remaining 4 items shows that they explain 75% of the variance with two factors extracted. All items in this analysis had factor loadings of >0.5 and communalities (extraction) of >0.25. The pattern matrix of the factor loading is presented in Table 4. Item no. 1 *(“Cognitive errors in general have important impact towards clinical decision making in emergency medicine”*) and item no. 2 *(“Being aware of cognitive errors help me to be more careful in my clinical decisions”*) load on Factor 2 whereas item no. 5 *(“The understanding of cognitive errors and its impact on clinical decision making and patient safety should be made a component in emergency medicine curriculum in postgraduate training”)* and item no. 6 *(“The understanding of cognitive errors and its impact on clinical decision making and patient safety should be taught at undergraduate level”)* load on Factor 1. Hence, we labeled factor 1 as the “educational interventions to reduce the risk of cognitive errors” whereas Factor 2 is labeled as the “impact of cognitive errors in clinical decision making”.

**Table 4:** Pattern Matrix of the Factor Loadings

	Factor	
	1 “Educational interventions to reduce the risk of cognitive errors”	2 “Impact of cognitive errors in clinical decision making”
Item no. 1 *“Cognitive errors in general have important impact towards clinical decision making in emergency medicine”*		0.616
Item no. 2 *“Being aware of cognitive errors help me to be more careful in my clinical decisions”*		0.590
Item no. 5 *“The understanding of cognitive errors and its impact on clinical decision making and patient safety should be made a component in emergency medicine curriculum in postgraduate training”*	0.668	
Item no. 6 “*The understanding of cognitive errors and its impact on clinical decision making and patient safety should be taught at undergraduate level”*	0.601	

Principal Axis Factoring was used as the extraction method

Promax oblique rotation with Kaiser normalization was used as the rotation method

Values of factor loading <0.5 are suppressed and not displayed

Both factors in Part A yield a Cronbach’s alpha of 0.676 and 0.635 respectively and no further improvement in the Cronbach’s alpha values could be achieved by deleting any of the items. Cronbach’s alpha for Part B is 0.657. Similarly, no further improvement in Cronbach’s alpha value could be achieved by deleting any of the items in this part. In the finalized version of the CATChES questionnaire (Table 5), the sequence of the factors is logically reversed, with Factor 2 placed first before Factor 1 for Part A of the questionnaire.

**Table 5:** The Final Version of the CATChES Questionnaire

Part A For this part, evaluate your response using the Likert scale where 1 = strongly disagree, 2 = disagree, 3 = neutral, 4 = agree, 5 = strongly agree					
Items	Likert scale				
Impact of cognitive errors in clinical decision making					
Cognitive errors in general have important impact towards clinical decision making in emergency medicine	ᅟ	ᅟ	ᅟ	ᅟ	ᅟ
	1	2	3	4	5
Being aware of cognitive errors help me to be more careful in my clinical decisions	ᅟ	ᅟ	ᅟ	ᅟ	ᅟ
	1	2	3	4	5
Educational interventions to reduce the risk of cognitive errors					
The understanding of cognitive errors and its impact on clinical decision making and patient safety should be made a component in emergency medicine curriculum in postgraduate trainingᅟ		ᅟ	ᅟ	ᅟ	ᅟ
	1	2	3	4	5
The understanding of cognitive errors and its impact on clinical decision making and patient safety should be taught in undergraduate level	ᅟ	ᅟ	ᅟ	ᅟ	ᅟ
	1	2	3	4	5
Part B For this part, state whether the following categories of cognitive errors are relevant in your clinical practice					
Cognitive errors due to over-attachment to a particular diagnosis	Relevant			Not relevant	
	ᅟ			ᅟ	
Cognitive errors due to failure to consider alternative diagnoses	Relevant			Not relevant	
	ᅟ			ᅟ	
Cognitive errors due to inheriting someone else’s thinking	Relevant			Not relevant	
	ᅟ			ᅟ	
Cognitive errors in prevalence perception or estimation	Relevant			Not relevant	
	ᅟ			ᅟ	
Cognitive errors involving patient characteristics or presentation context	Relevant			Not relevant	
	ᅟ			ᅟ	
Cognitive errors that are associated with the doctor’s affect or personality	Relevant			Not relevant	
	ᅟ			ᅟ	

## Discussion

Based on the content validity evaluation of Part A and B of the questionnaire, all items were retained as they were shown to have excellent content validity in terms of their relevance in clinical setting.

From the EFA, Part A of the questionnaire is constructed with two factors, i.e., the “impact of cognitive errors in clinical decision making” and “educational interventions to reduce the risk of cognitive errors”. Each of these two factors has two items. Referring back to the Transtheoretical Model of Change by Prochaska et al. [9, 10], Factor 2 “impact of cognitive biases in clinical decision making” reflects the contemplation stage of the model, whereas Factor 1 “educational interventions to reduce the risk of cognitive biases” reflects the preparation stage of the model.

Furthermore, from the EFA, it is also shown that there are two items that had to be removed. For item no. 4 *(“Something, rather than nothing, can be done to minimize the risk of falling into these biases”)*, the phrase ‘something, rather than nothing’ is rather ambiguous and this may have resulted in its rejection by the participants as a valid item. Re-phrasing it with a more direct sentence may bring greater clarity. For example, it could be rephrased, as ‘Specific de-biasing strategies are effective in minimizing the risk of committing cognitive biases.

For item no. 3 *(“Authority gradient discourages critical thinking and thus increase the vulnerability to commit cognitive errors”)*, its rejection could be due to the fact that the statement is overly generalized, particularly in an Asian culture. Authority gradient is defined as the gradient that may exist between two individuals’ professional status, experience, or expertise that contributes to gap in exchanging information or communicating concerns [17]. In our study, perhaps our participants did not think that authority gradient is always bad. Nurtured in an environment where a healthy level of authority gradient is respected, a senior, experienced clinician can train a junior clinician in inculcating better clinical decision making skill.

In terms of the internal consistency analysis of Part A, a moderate degree of internal consistency measured by both Cronbach’s alpha values of more than 0.6 in both factors was noted. The internal consistency could be improved by adding more items within the factors. Therefore, future research should consider including more items that are relevant to Factor 1 (educational interventions to reduce the risk of cognitive biases) and Factor 2 (impact of cognitive biases in clinical decision making) or items to generate more factors to move up to the next of stage of “taking actions” along the ladder of Transtheoretical Model of Change [9].

There are a number of limitations in this validation study. First, for Part A, confirmatory factor analysis (CFA) was not performed on another set of samples to confirm the constructs developed based on the EFA result. Second, face validity was not performed to determine its comprehensibility and readability. For example, as mentioned, the phrase ‘something, rather than nothing’ in item no. 4 is rather vague. Third, more items should be included to improve the internal consistency of the constructs. The future development of this project would include rewording and rephrasing the items as well as adding more relevant items based on Transtheoretical Model of Change [9]. More samples should be included to replicate the present EFA results and as well as including CFA test in the analysis to devise the second edition of this questionnaire.

## Conclusion

Despite its limitations, the construct and content validation suggest that the CATChES questionnaire tool is useful in evaluating the awareness among clinicians toward cognitive errors in clinical decision making. Such awareness may in turn, motivate them to take measures to minimize risk of committing these errors.

## References

[1] National Academies of Sciences, Engineering, and Medicine (2015). Improving diagnosis in health care.

[2] Graber ML, Franklin N, Gordon R (2005). Diagnostic error in internal medicine. Arch Intern Med.

[3] Berner ES, Graber ML (2008). Overconfidence as a cause of diagnostic error in medicine. Am J Med.

[4] Croskerry P (2003). The importance of cognitive errors in diagnosis and strategies to minimize them. Acad Med.

[5] Croskerry P, Singhal G, Mamede S (2013). Cognitive debiasing 1: origins of bias and theory of debiasing. BMJ Qual Saf.

[6] Bornstein BH, Emler AC (2001). Rationality in medical decision making: a review of the literature on doctors’ decision-making biases. J Eval Clin Pract.

[7] Klein JG (2005). Five pitfalls in decisions about diagnosis and prescribing. BMJ.

[8] Campbell SG, Croskerry P, Bond WF (2007). Profiles in patient safety: a “perfect storm” in the emergency department. Acad Emerg Med.

[9] MacDonald OW. Physician Perspectives on Preventing Diagnostic Errors. 2011. In: QuantiaMD. Available at URL: https://www.quantiamd.com/q-qcp/QuantiaMD_PreventingDiagnosticErrors_Whitepaper_1.pdf, Accessed 23 Oct 2015.

[10] Prochaska JO, DiClemente CC, Norcross JC (1992). In search of how people change. Applications to addictive behaviors. Am Psychol.

[11] Chew KS, van Merrienboer J, Durning S. Teaching cognitive biases in clinical decision making: a case-based discussion. MedEdPORTAL Publications. 2015;11:10138. http://dx.doi.org/10.15766/mep_2374-8265.10138

[12] Lynn MR (1986). Determination and quantification of content validity. Nurs Res.

[13] Hair JF, Black WC, Babin BJ, Anderson RE (2010). Multivariate data analysis.

[14] Kline RB (2011). Principles and practice of structural equation modeling.

[15] Cho E, Kim S (2015). Cronbach’s coefficient alpha: well known but poorly understood. Organ Res Methods.

[16] Polit DF, Beck CT, Owen SV (2007). Is the CVI an acceptable indicator of content validity? Appraisal and recommendations. Res Nurs Health.

[17] Cosby KS, Croskerry P (2004). Profiles in patient safety: authority gradients in medical error. Acad Emerg Med.

